# The role of the harmonic vector average in motion integration

**DOI:** 10.3389/fncom.2013.00146

**Published:** 2013-10-21

**Authors:** Alan Johnston, Peter Scarfe

**Affiliations:** ^1^Department of Cognitive, Perceptual and Brain Sciences, University College LondonLondon, UK; ^2^CoMPLEX, University College LondonLondon, UK; ^3^Department of Psychology, School of Psychology and Clinical Language Sciences, University of ReadingReading, UK

**Keywords:** global motion, plaids, motion computation

## Abstract

The local speeds of object contours vary systematically with the cosine of the angle between the normal component of the local velocity and the global object motion direction. An array of Gabor elements whose speed changes with local spatial orientation in accordance with this pattern can appear to move as a single surface. The apparent direction of motion of plaids and Gabor arrays has variously been proposed to result from feature tracking, vector addition and vector averaging in addition to the geometrically correct global velocity as indicated by the intersection of constraints (IOC) solution. Here a new combination rule, the harmonic vector average (HVA), is introduced, as well as a new algorithm for computing the IOC solution. The vector sum can be discounted as an integration strategy as it increases with the number of elements. The vector average over local vectors that vary in direction always provides an underestimate of the true global speed. The HVA, however, provides the correct global speed and direction for an unbiased sample of local velocities with respect to the global motion direction, as is the case for a simple closed contour. The HVA over biased samples provides an aggregate velocity estimate that can still be combined through an IOC computation to give an accurate estimate of the global velocity, which is not true of the vector average. Psychophysical results for type II Gabor arrays show perceived direction and speed falls close to the IOC direction for Gabor arrays having a wide range of orientations but the IOC prediction fails as the mean orientation shifts away from the global motion direction and the orientation range narrows. In this case perceived velocity generally defaults to the HVA.

## Introduction

The first stage of motion analysis is not a global estimate of the motion of an object as such, but a dense set of estimates of the motion present at each location in the visual field. These local estimates need to be grouped and combined to determine the motion of the object as a whole. However, this local analysis can be prone to the aperture problem (Wallach, 1935). The aperture problem results from the redundancy inherent in a 1-dimensional pattern, like a line or sine grating, embedded in a 2-dimensional space. The true 2-dimensional velocity of an infinite line cannot be determined. Neurons in the early part of the visual system have small receptive fields. Contours for which the variation in orientation is small relative to the aperture of a receptive field will appear approximately 1-dimensional. In the case of a 1-dimensional pattern viewed through an aperture, human observers typically see motion in the direction orthogonal to the contours (Wallach, 1935; Hildreth, 1984).

Strategies for combining motion estimates were initially introduced to explain the neural computation of pattern motion in plaids, which are formed from the superposition of two 1-dimensional gratings (Adelson and Movshon, 1982). It was proposed that the grating components are initially processed independently following orientation filtering by the visual system and then the resulting velocities are combined. Each component constrains the possible 2-dimensional pattern motion but a unique velocity could be arrived at as the velocity that satisfies both constraints. This two-stage strategy is generally referred to as the intersection of constraints (IOC) solution (Adelson and Movshon, 1982).

However, a number of studies have shown that the ideal IOC solution for plaids does not accurately reflect perceived velocity. The plaid perceived direction is biased toward the high contrast component although the IOC computation depends only upon velocity (Stone et al., 1990; Champion et al., 2007). Manipulations that would have been expected to alter the perceived speed of one of the components, such as reducing its contrast (Stone et al., 1990), altering spatial frequency (Smith and Edgar, 1991) or adapting to motion in one component direction (Derrington and Suero, 1991) shift the apparent direction of the plaid in the direction that is consistent with the application of the IOC principle to the perceived motion of the components. However, changes in perceived speed of components prior to an IOC computation cannot explain all effects of changing component characteristics on the perceived direction of plaids (Champion et al., 2007).

Ferrera and Wilson (1990) showed that if the grating components were similar in direction and both moved to the right or left of the IOC direction (type II plaids), rather than straddling the IOC direction (type I plaids), then the perceived direction of the plaid was biased by an average of around 7.5 degrees toward the vector sum of the components. At short durations, ≤60 ms, Yo and Wilson (1992) reported that plaids were seen to move in the vector sum direction but shifted toward the IOC direction at longer durations. However, Bowns (1996) showed that whether movement in the vector sum direction was seen or not at short durations depended upon the component combination used in the plaids. Some component combinations led to local motion ambiguities in the plaid, which Bowns observed arose from the motion of edge features in the vector sum direction and blob features in the IOC direction. Bowns and Alais (2006) showed that adaptation to grating motion in the vector average (VA) direction shifted the perceived motion toward the IOC direction and *vice versa*, indicating the balance between these alternatives could be altered.

There has been less emphasis on plaid speed perception than direction perception. Ferrera and Wilson (1991) reported that the perceived speed of type I plaids was underestimated when compared to a sine grating that had the same spatial frequency as the components but matched the IOC speed when compared to a sine grating whose period matched the period of the plaid nodes. Castet and Morgan (1996) also showed an underestimate of IOC speed for a plaid with constant component speed whose pattern speed increased as the angle between the components increased.

Approaches to plaid motion recovery include the analysis of the activity of a population of spatio-temporally tuned filters (Simoncelli and Heeger, 1998), direct computation from spatio-temporal gradients (Weiss et al., 2002; Johnston et al., 2003; Dimova and Denham, 2009) and the encoding of the spatio-temporal pattern of tracked features (Bowns, 2011). However, some of these strategies for recovering the direction are highly contextualized by the characteristics of plaids. The assumption of the independent analysis of components requires unrealistically narrow spatiotemporal filtering, particularly with respect to temporal frequency since there are only two or three temporal mechanisms spanning the temporal domain (Snowden and Hess, 1992; Johnston and Clifford, 1995). Spatiotemporal gradient approaches to computing pattern motion are more generic and do not make any special reference to the content of the image.

The global Gabor array (Amano et al., 2009; Rider et al., 2009; Scarfe and Johnston, 2010, 2011) provides a simpler paradigm in which to study the integration of local motion signals. In this case, as in the case of windowed line motion (Mingolla et al., 1992), the local spatial pattern is essentially 1-dimensional, and integration necessarily occurs over space rather than potentially at a single point in space.

**Figure 1:**
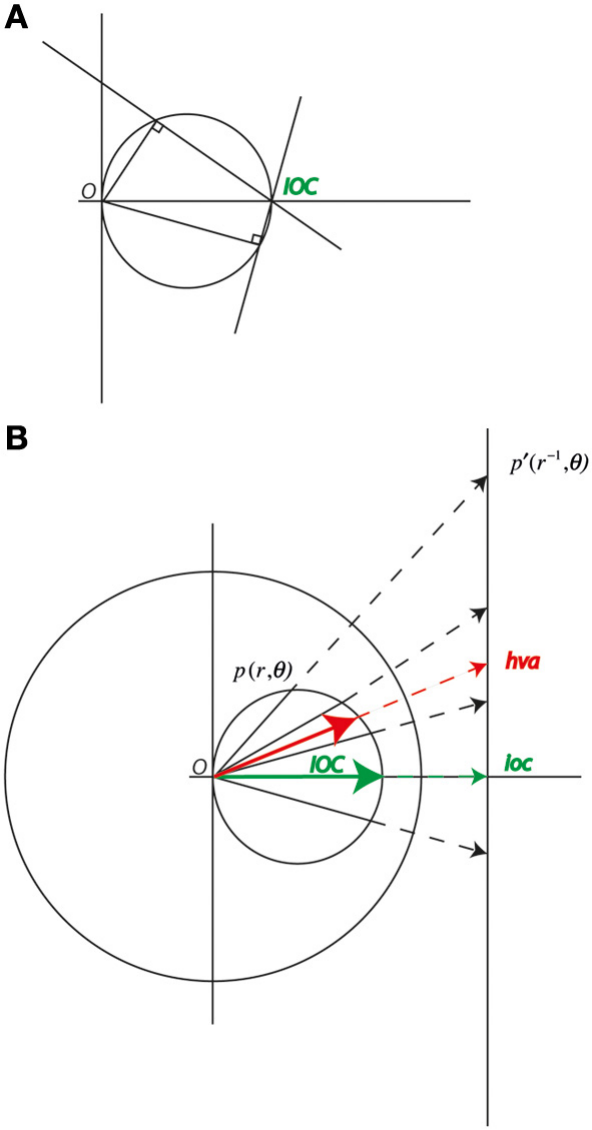
**(A)** A velocity space diagram with vectors representing two component normal velocities and constraint lines representing global motion vectors that are consistent with the normal component. The constraint lines for any two vectors lying on the same circle through velocity space will intersect at a single point representing the global motion. **(B)** Points on a circle through the origin inverted in the unit circle about the origin project to a straight line in velocity space. The inverse of the global motion (ioc) can be found as the vector that minimizes the variation in the magnitude of the components of the projection of the sample vectors onto this as yet undetermined vector. The global motion (IOC) is the inverse of ioc. Note the average of any of the sample vectors (hva) must lie on the line though the samples and the ioc, and its inverse, the harmonic vector average (HVA), must lie on the circle through the origin.

A rigidly moving object generates a characteristic distribution of normal velocities. The magnitudes of the local speeds are the global speed times the cosine of the angle between the normal component of contour motion and the global direction of motion of the object (Figure 1A). Taking a cue from this, a pattern of local motion can be generated by an array of Gabor patches, which consist of moving sine gratings windowed by a Gaussian. When the speed of the grating motion is set to conform to the pattern for a rigidly moving contour, i.e., to be some global speed times the cosine of the angle between the normal component of the sine grating motion and the global motion direction, the array of patches typically appear to cohere into a single moving surface (Amano et al., 2009).

The global percept can break down when the elements can be grouped on the basis of carrier spatial frequency and the carrier frequencies are very different (Maruya et al., 2010). However, in general, Gabor arrays appear to move approximately in the IOC direction with the implied global speed. Arrays of plaid elements with the same pattern of velocities but in which the local velocity is well defined and unambiguous appear to have a direction and speed consistent with the VA rule (Amano et al., 2009). However, this is not the only way of combining vectors and other alternatives should be considered. The natural alternative to the IOC result is not the vector sum or VA but the harmonic vector average (HVA), which provides a way of aggregating local velocities that takes into account the aperture problem. In the HVA the directions of the vectors are unchanged but their magnitudes are inverted. The magnitude of a vector is inverted by dividing it by its squared length. The harmonic average is often used to average rates, particularly when the variable is on the denominator (Ferger, 1931) e.g., averaging speeds over the same distance. In our case the HVA arises naturally from the geometry of the velocity space diagram (Figure 1). The HVA is not to be confused with the inverse-vector-average rule (Verstraten et al., 1994; Alais et al., 2005), which describes how the direction of the transparent motion aftereffect is accounted for by a VA in which component directions are weighted according to the sensitivity (inverse threshold) or duration of the component MAEs.

We argue below that, if the aim of a vector combination calculation is to tell us something about the global motion of an object, where the individual measures are ambiguous reflecting the aperture problem, then the VA has little or no merit, since it does not provide a useful measure of the speed of the global motion. We will see that the HVA does carry useful information about the global speed. The VA may, however, reflect the operation of the visual system in recovering summary statistics of velocity fields.

### Theory

Although the underlying geometry of the IOC rule indicates it should be possible to solve for the IOC velocity, given the component velocities in the case of plaids or two normal component velocities in the case of a pair of Gabor elements, there is no consensus on the algorithm the brain might use to compute the IOC. Given a description of the constraint lines the IOC can be calculated by the method described by Bowns (1996). Here we revisit the velocity space geometry to describe a simple means of computing the global velocity (as in the IOC strategy) from the sample velocities, which does not explicitly represent the constraint lines or their intersections (Jasinschi et al., 1992) and which readily accommodates multiple samples. We also introduce the HVA, which provides a viable alternative combination rule for local 1d signals.

Figure 1B shows a range of potential normal component velocities for a single global velocity. All these velocities lie on a circle through the origin. The inversion of a point *p*(*r*, ϑ) in the unit circle is the point *p*′(*r*^−1^, ϑ). The inversion of a circle passing through the origin is a straight line (Brannan et al., 1999). The IOC solution derives from the vector, which minimizes, in a least squares sense, the variation in the projections of a set of sample vectors onto it. The IOC solution is

(1) $$IOC={\left(\begin{array}{c} u\;\cdot \;u\;v\;\cdot \;u\\ u\;\cdot \;v\;v\;\cdot \;v \end{array}\right)}^{-1}\left(\begin{array}{c} u\;\cdot \;n\\ v\;\cdot \;n \end{array}\right),$$

where **u** and **v** are the vectors of the Cartesian components of *p*′(*r*^−1^, ϑ). The HVA is

(2) $$HVA=\frac{k}{{\left|\left(\begin{array}{c} \sum u\\ \sum v \end{array}\right)\right|}^{2}}\;\left(\begin{array}{c} \sum u\\ \sum v \end{array}\right),$$

where *k* is the number of motion samples.

Therefore

(3) $$IOC={\left(\begin{array}{c} u\;\cdot \;u\;v\;\cdot \;u\\ u\;\cdot \;v\;v\;\cdot \;v \end{array}\right)}^{-1}{\left|\left(\begin{array}{c} \sum u\\ \sum v \end{array}\right)\right|}^{2}k^{-1}\;HVA$$

See Appendix for more detail.

The vector sum and VA of do not provide a useful combination rule for the computation of global speed in multiple component plaids or Gabor arrays. The vector sum increases with the number of elements and the average velocity tends to a value that is half the speed of the object motion as the range of orientations increase (see Figure 1). The HVA of a set of velocity samples on the other hand lies on the circle through the origin in velocity space that describes the range of possible normal components. Because of this property the HVA could be used for iterative spatial averaging since the intermediate means could be the normal components of some contour. The HVA aggregated estimates could therefore be input to a further HVA or IOC computation without loss of fidelity. Note the HVA, as expressed above requires the number of samples, since it is an average, however, the 4 IOC algorithm does not require this information.

The HVA could provide an excellent proxy for an explicit IOC global motion computation so long as the average orientation of the normal components of the local motion is unbiased relative to the global direction of motion. The IOC calculation will give the correct global velocity for a Gabor array irrespective of whether the samples straddle the correct global velocity (type I arrays) or whether they are constrained to lie on one or other side of the correct global velocity (type II). However, for type II arrays the VA and the HVA calculation will differ from the IOC velocity. Amano et al. (2009) found that for short durations a type II Gabor array with two orientations appeared to move in the VA direction for short durations and appeared to move in the direction of the IOC solution at longer durations. They did not consider the HVA but the directions of the VA and the HVA will be similar. We wanted to determine whether the IOC or HVA best predicted perceived speed and direction of global motion and therefore measured perceived velocity for a range of type I and II Gabor arrays. In the first experiment we measured perceived direction for type I and II arrays with different distributions of Gabor orientations. We then measured perceived speed for these arrays to determine the degree to which perceived global velocity followed the IOC or HVA solution. We found perceived velocity was close to the IOC prediction for arrays with a wide range of orientations. The IOC prediction fails as the mean orientation shifts away from the global motion direction and the orientation range narrows. In this case perceived velocity tends to default to the HVA.

## Materials and methods

Stimuli were displayed on a 20 inch CRT monitor (Mitsubishi Diamond Plus 230B), with a 1024 by 864 pixel resolution and refresh rate of 85 Hz. The monitor was gamma corrected and the pixels were square. Observers were positioned in a head and chin rest. The viewing distance to the screen was 80cm and a normal projected from the midpoint of the monitor screen intersected the cyclopean eye. At this viewing distance the monitor subtended approximately 28 by 21 degrees. The stimuli were rendered online in Matlab using the Psychophysics Toolbox extensions (Brainard, 1997; Kleiner et al., 2007).

### Participants

In total five participants took part in the experiment. Three were experienced psychophysical observers, including the author PS. All bar PS were completely naïve as to the purposes of the experiment.

### Stimuli

**Figure 2:**
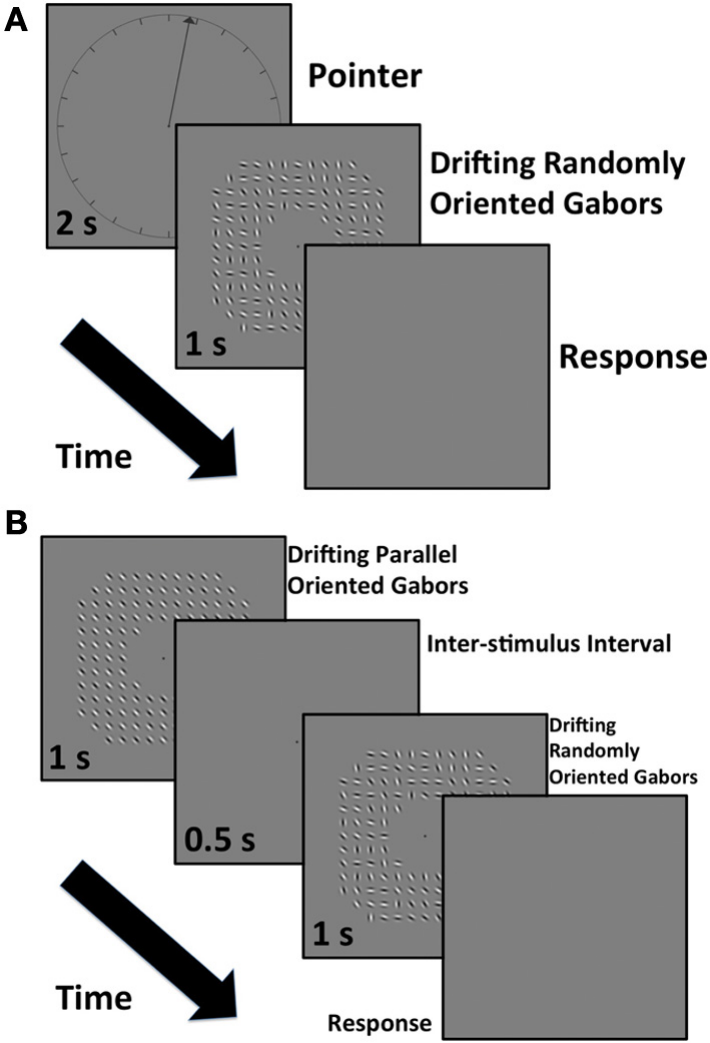
**(A)** The experimental time sequence for measurement of perceived direction. **(B)** The experimental time sequence for the measurement of perceived speed.

An example of the annular dynamic Gabor arrays used in the experiment is shown in Figure 2. The Gabor elements have been expanded in the figure to increase visibility. The annulus was generated by first defining a 27 × 27 grid of Gabor elements. The Gabor elements were constructed as Gaussian windowed sine gratings. Each Gabor subtended 0.5 degrees of visual angle with a spacing of 0.1 degree around all sides. Each Gabor had a 50% contrast carrier with a spatial frequency of 4 cycles per degree, which was windowed through multiplication by a Gaussian with a sigma value of 0.1 degrees. The square grid subtended 16.1 degrees of visual angle and was centered on the screen. To make the annular stimulus any Gabor whose center was outside an outer circle of 9 degrees or within an inner circle of 3.3 degrees was removed from the grid.

## Results

### Experiment 1 (direction)

In Experiment 1, observers judged the direction in which the Gabor array appeared to move. The drift rate of the individual Gabors in the array was set relative to a 2d velocity of 0.75 cycles per second in an upward direction (here referenced as zero degrees). We refer to this as the global motion speed and direction. The drift speeds of each element was the cosine of the difference in angle between the normal velocity component of the Gabor and the global motion direction,

$$S_{C}=S_{G}\;cos\;\left(\vartheta -\vartheta_{G}\right)a$$

where ϑ_*G*_ is the global direction, *S*_*G*_ is the global motion speed, ϑ is the orientation of the local normal to the carrier of an individual Gabor element and *S*_*C*_ is the drift speed it needs to be consistent with the global motion drift velocity (Adelson and Movshon, 1982; Amano et al., 2009).

In Experiment 1 we measured the effect of varying the distribution of Gabor angles in the array on the perceived global motion direction. We varied the mean angle of the Gabors and the degree of variation around this value whilst holding the global motion direction and global motion speed constant. The centers of the range were 0, 11.25, 22.5, 33.75, and 45 degrees (positive values being in a clockwise direction), and the extents of the ranges were 30, 50, 70, and 90 degrees. As an example, for a mean of 45 degrees and a range of 90 degrees the angles for the Gabors were randomly chosen from a uniform distribution between 0 and 90 degrees. A new set of Gabor angles was generated from each distribution on each trial. The phase of each Gabor was also randomized on each trial.

Overall there were 20 blocks of trials - 5 range centers by 4 ranges. These were completed in a randomized order for each observer. In order to measure the perceived direction of the array we adopted a binary choice design. In the first interval the observers were presented with a clock face with a radius of 9.6 degrees centered on the screen (Figure 2A). The clock face was marked at 15-degree intervals around its edge with internal radial line segments abutting the circle, which were 0.74 degrees in length. An arrow pointing out from the center of the clock indicated a direction against which the observer was asked to compare the direction of movement of the array in the second interval. The clock face and arrow were presented for 2 s. In the second interval the drifting Gabor array was presented for 1 s. The screen was then set to the mean gray value and the observer had to indicate whether the Gabor array appeared to move clockwise or counter-clockwise of the direction indicated by the previously seen arrow. We varied the direction of the arrow on each trial to generate a psychometric function. There were seven arrow directions and each was presented in a randomized order 20 times in a block. The exact values depended on the block type and the observer. We fitted a cumulative Gaussian to the observer's data and determined the point of subjective equality (PSE) and 95% confidence intervals around this value in Matlab using a bootstrapping technique (Wichmann and Hill, 2001a,b). The PSE represents the perceived angular direction of motion of the Gabor array.

**Figure 3:**
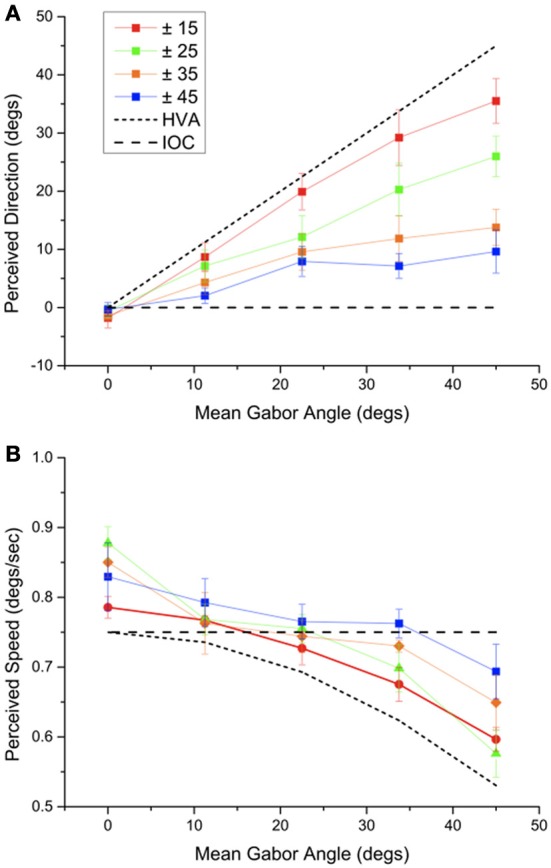
**(A)** The apparent direction of a Gabor array with a particular mean motion direction and range of orientations. **(B)** The apparent speed of a Gabor array with a particular mean motion direction and orientation range measured using an array of parallel Gabor elements moving in the apparent motion direction (as measured in **A**). Error bars show ± 1 s.e.

Figure 3A shows perceived direction of motion averaged across our five observers for each type of array. Error bars show one standard error of the mean. The diagonal line shows the prediction for the HVA the prediction for the IOC is 0 degrees, upwards, in each case. It is clear that, as the direction range decreases, the perceived direction shifts from the IOC direction toward the HVA direction. However, the perceived direction is in all cases, other than when the average direction is upwards, biased in the direction of the mean motion direction. Also even for the narrowest range the perceived direction is slightly biased in the IOC direction.

We can ask, if the perceived direction is not in the IOC direction is the perceived speed consistent with the change in perceived direction of motion?

### Experiment 2 (speed)

In Experiment 2 we measured the perceived speed of each of the global motion Gabor arrays used in Experiment 1 using the method of constants. In order to measure the speed of global motion in the perceived direction of motion we set the direction of the standard array individually to the apparent direction for each subject and condition measured in Experiment 1. The standard array was an array of Gabors all orientated orthogonal to the global motion direction (Figure 2B). We also refer to this as the parallel-orientated array. This array was presented first for 1 s.

After a half second gray interval with only the fixation point present, a second comparison array was presented for 1 s. The comparison arrays had the same parameters as those in Experiment 1 i.e., Gabors for the arrays were selected from uniform distributions with ranges of 30, 50, 70, and 90 degrees centered on either 0, 11.25, 22.5, 33.75 or 45 degrees. The phase of each Gabor was randomized on each trial. The comparison array was drifted at one of 7 different speeds and each speed was presented 20 times, randomly ordered across trials within a block. Blocks were completed in a randomized order for each observer. The observers' task was to report which array drifted faster. We fitted a cumulative Gaussian to the observers' data and determined the PSE and 95% confidence intervals around this value in Matlab using a bootstrapping technique (Wichmann and Hill, 2001a,b). The PSE provided a measure of the perceived speed of each Gabor array. Examples of the stimuli presented are available as supplementary information.

**Figure 4:**
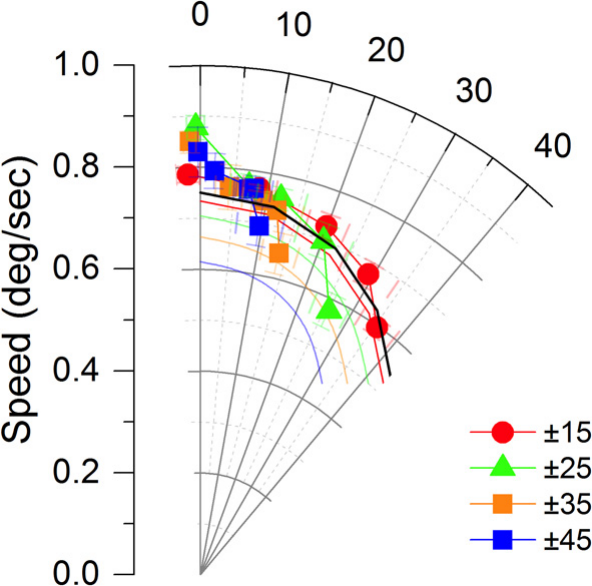
**The perceived speed and direction of the global motion from Figure 3 combined to show perceived velocity.** The global motion has a speed of 0.75 degrees/ sec and a direction of 0 degrees in this representation. The curved axes shows degrees of visual angle from the global motion direction. The lines without symbols give the predictions for the vector average for the different direction ranges and mean directions. Error bars show ± 1 s.e.

Figure 3B shows perceived speed across our five observers for each type of array. Error bars show the standard error of the mean. Perceived speed was close to the IOC speed for the arrays with a greater range of orientations but as the mean local direction shifted away from the global motion direction and the orientation range narrowed the perceived velocity came closer to the HVA. To determine whether perceived speed and perceived direction co-varied systematically, as would be expected from a HVA calculation, we plotted perceived speed and direction in a velocity space plot (Figure 4). It is clear that most of the points lie close to the IOC prediction. When the orientation range is reduced the IOC prediction fails and the data fall close to the HVA prediction shown in black. The perceived speed is overestimated when the directions of motion in the Gabor array are uniformly distributed around the vertical. Perceived speed appears to be underestimated, falling below that of the predicted HVA in two conditions in which the mean orientation is ± 45 degrees from the global motion direction. The colored lines without symbols give the predictions for the VA calculation. For a narrow range of orientations the predictions for the VA and the HVA are similar, however, for a broad range of orientations the data favor the HVA prediction.

We can draw a number of conclusions on the basis of this data. The shift in apparent direction away from the HVA direction (Figure 4) toward the global motion direction cannot be explained on the basis of the HVA calculation alone. This shift is consistent with the IOC computation but when the IOC computation fails the perceived speed and direction of motion vary together in a way that is predicted by the HVA not the IOC. The perceived speed only drops below the HVA prediction for the largest difference between the true global motion direction and the mean local motion direction.

## Discussion

The global Gabor array simplifies the problem of investigating motion integration since, unlike plaids or more complex spatial patterns, we do not need to assume Fourier components are analyzed separately and there are no features arising from their combination moving in the global motion direction that could be tracked (Mingolla et al., 1992). However, combination rules such as the IOC rule and motion vector combination rules, developed to explain 2d plaid motion might still apply. The IOC solution and the HVA, introduced here, predict the same global motion for type I Gabor arrays in which there is an unbiased distribution of motion directions around the global motion direction. Although the vector sum and the VA can give a good estimate of the direction of global motion, they do not provide a correct measure of the global speed for Gabor arrays containing a range of Gabor orientations, or generalizing from this, object motion. We can discount the vector sum as a way of combining multiple velocity samples, as the value of the sum clearly increases with the number of samples. The VA provides a measure of global motion, which is half the speed of the true motion for a uniform distribution of contour orientations covering the full range of possible contour orientations, although the VA and HVA predictions converge as the orientation range narrows.

The HVA is to be preferred to the VA for a number of reasons. Firstly it provides the correct global velocity for an unbiased distribution of oriented contours relative to the global motion direction. This, in addition, means the HVA gives the correct global velocity for a moving simple closed contour. It is clear from symmetry that the integrated normal components along a hemi-circular contour joining two points with tangents parallel to the flow will be in the direction of the global motion vector. Now consider a uniform flow field and a more general smooth contour joining points with tangents parallel to the flow. The integral of the projection of the flow on the outer normal to the contour along a simple closed contour gives the flux of the motion vector field through the contour. This scalar value can be interpreted as the amount of material flowing over the contour. This is same as the line integral of the normal component speeds along the leading contour for a contour moving at a constant velocity. Since for a uniform motion field the flux, the amount of material flowing over the contour, does not depend upon the path of the contour (as we have described it), the flux will be the same as for the hemi-circle. Thus, in both cases the normals pointing to either side of the global flow must be balanced and the average motion vector, and hence the average inverse motion vector, will be in the direction of the global motion. The same logic applies to the trailing contour with forward facing normals. This allows us to conclude that for a simple closed contour (or such arising from a level set of the image brightness) there is an unbiased distribution of oriented contours and the HVA over the contour gives the global object speed and direction.

Secondly, for a limited range of orientations, as might be found in a local region, the HVA provides the best way of aggregating 1d information about global velocity for that region, since it takes into account the aperture problem in combining normal components. Even in the case of a biased distribution, the HVA can serve as a valid intermediate measure, which can subsequently be combined with other local HVAs in a new HVA or be combined through an IOC combination to give the correct global velocity. The VA does not have this property. Once a VA (over different directions) is formed it cannot be combined with other local vector averages or with a local normal component to give the correct global motion. Grouping local motion vectors through the HVA can therefore improve signal to noise by averaging, without corrupting the signal. An additional advantage of a noise-reducing high fidelity intermediate local calculation is that it can serve global motion computations that are more complex than translation such as rotation and expansion (Lee and Lu, 2010). The HVA also gives appropriate weight to small speeds in the global computation, such as those arising from contours oriented away from the global motion direction, which would not contribute much to a VA.

The principle underlying both the IOC algorithm and the HVA algorithm is that the local velocities reflect the normal components of the motion of an object generated by a single motive cause, namely translation. If the local motion is unambiguous, even if the pattern of velocities is the same, there is no reason to assume a single underlying cause as each local motion vector may correspond to the movement of a single particle. In this case the motion will not appear to cohere and alternative strategies for reporting on the population of local velocities might apply, including the VA as a summary statistic. Amano et al. (2009) compared the perceived speed of Gabor arrays with plaid arrays in which the unambiguous motion and direction of the plaids matched the normal components of motion of the Gabor elements in a Gabor array. They reported the perceived motion of the plaid array was slower than the Gabor array and that it approached the VA speed. Amano et al. conclude that the motion system can group flexibly - grouping ambiguous signals by IOC and averaging unambiguous local signals by VA. Amano et al. report that this plaid array did not cohere, which highlights the distinction between grouping to a single solution and a summary statistic of a space-variant array of velocities. A lack of coherence may also explain why perceived speed did not reach the HVA speed in some of our conditions (see supplementary material).

The HVA and the IOC approach outlined here might also be applied to the integration of component velocities in plaid patterns. However, the key issue in extending the current approach is whether plaid components are separated and analyzed independently by the visual system. Recent work showing V5/MT cells differ in their response to pseudo plaids in which two non-overlapping Gabor patches are presented to the visual field and plaid patches in which the components overlap (Majaj et al., 2007) suggest that 2d patterns are processed differently to multiple 1d patterns. In addition the component approach has difficulty accounting for the Amano et al. (2009) result, that there is a difference in motion coherence threshold for arrays in which plaids elements indicating a single global velocity are combined with plaid noise elements, as compared to when components from these two types of array elements are mixed to give a variety of local plaid velocities (Amano et al., 2009). This suggests 2d motion is computed locally from the 2d image rather through the combination of 1d components.

There is a greater tendency to see motion in the VA or equivalently the HVA direction when stimulus presentations are short (Yo and Wilson, 1992). The harmonic vector sum appears in both the HVA and IOC algorithms. One possibility is that the harmonic vector sum is computed first, which would give an indication of the (incorrect) global motion direction, and that the computation of the correct speed and direction follows after a process of refinement. This could occur through local HVA computations feeding into a global IOC calculation. Although the IOC can readily be computed in type II arrays the global motion direction is almost always biased in the HVA direction.

Weiss et al. (2002) were able to account for a number of psychophysical results, primarily relating to perceived direction. They extended the Lucas and Kanade (1981) method for computing velocity by adding a parameter to the leading diagonal of the matrix of the summed products of derivatives – sometimes referred to as the structure tensor (see their Equation 1). Without this addition to the leading diagonal, the matrix cannot be inverted for 1d moving pattern (lines, gratings etc.), since there is no unique solution – a reflection of the aperture problem. This method of ensuring a solution is referred to as ridge regression in the statistics literature (Hoerl and Kennard, 1970). The addition in Weiss et al. is motivated by a Bayesian argument. The value of the parameter is the ratio of the variance in the likely velocity, which is dependent upon image brightness measurement noise, and the variance of a slowness prior probability. It is assumed that this parameter differs between observers allowing the fitting of different values of this parameter across experiments. The velocity calculation will be most accurate for a close-to-uniform prior, since this minimizes the value to be added. As the prior probability distribution places greater emphasis on slowness, the computed speed will reduce, since probabilities are positive and the determinant of the structure tensor will increase. Solving for the velocity involves inverting the matrix, which entails dividing by the determinant. Weiss et al also applied their approach to velocity aggregation over space. They showed for a moving rhombus the perceived direction could be accounted for by their model, however, they did not investigate perceived speed for this experimental paradigm. In general, since the perceived velocity depends upon the likelihoods (spatially overlapping the constraint lines) summed across space this approach predicts perceived speed and direction would be close to the VA speed and direction, or slower, due to the slowness prior.

The HVA approach implies that some neurons in the primate visual system may encode inverse speed or slowness. There is in fact considerable evidence for MT/V5 neurons that reduce their firing rate as speed increases (Mikami et al., 1986; Rodman and Albright, 1987; Lagae et al., 1993; Palanca and Deangelis, 2003; Nover et al., 2005). These neurons have not previously been attributed a particular functional role, apart from perhaps signaling slowness as part of a population code, however, they could form part of the substrate of the HVA computation. The benefits of coding inverse speed in a gradient model of motion computation and the methods by which is can be computed has been outlined in some detail elsewhere (Johnston et al., 1999a,b, 2003).

## Conclusions

The HVA has not previously been considered as a way of aggregating visual local motion estimates. However, the HVA appropriately combines normal component velocities that are subject to the aperture problem. This can clearly be seen from a consideration of the underlying inversive geometry within velocity space. The correct global motion vector for type II arrays can only be arrived at though an IOC or equivalent calculation. However, the global motion solution is also arrived at more efficiently in the inverse space. Perceptually, when the IOC prediction fails, the global motion percept tends to the HVA. This indicates that shifts in perceived direction and speed are linked.

### Conflict of interest statement

The authors declare that the research was conducted in the absence of any commercial or financial relationships that could be construed as a potential conflict of interest.

## Supplementary material

Illustration of the type of stimuli used in the experiments.

1) movieMGA45R15: A movie sequence showing a dynamic Gabor array with a mean angle of 45 degree and Gabor orientation draw from a range ±15 degrees.

2) movieMGA45R45: A movie sequence showing a dynamic Gabor array with a mean angle of 45 degree and Gabor orientation draw from a range ±45 degrees.

## Appendix

Let us describe, in velocity space, a set of *k* local velocity vectors, **p**, arising from the rigid motion of an object, with Cartesian components (*u*_i_^−1^, *v*_i_^−1^), and a global vector, the IOC solution, **IOC**, which we want to find (Figure 1B). The vector **p**′ is the set of inversions in the unit circle of the vectors in **p**, with components (*u*_i_, *v*_i_). We can write an over determined set of equations each of which expresses the relation that the inner product of **ioc** with each of the local velocity vectors in **p**′ is equal to one. The inner product of each *p*′_*i*_ with **ioc** gives the component of the sample vector in the **ioc** direction. The **ioc** vector has the squared magnitude |**ioc**|^2^. Therefore, equating the inner products to one, *n*_*i*_ = 1, has the effect of inverting the **ioc** vector (dividing **ioc** by |**ioc**|^2^) to give the **IOC** vector which lies on the circle through the origin.

(1) $$\begin{array}{l} {p^{\prime }}_{1}\cdot IOC=n_{1}\\ {p^{\prime }}_{2}\cdot IOC=n_{2}\\ \vdots \\ {p^{\prime }}_{k}\cdot IOC=n_{1} \end{array}$$

solving for **IOC** we have

(2) $$IOC={\left(\begin{array}{c} u\;\cdot \;u\;v\;\cdot \;u\\ u\;\cdot \;v\;v\;\cdot \;v \end{array}\right)}^{-1}\left(\begin{array}{c} u\;\cdot \;n\\ v\;\cdot \;n \end{array}\right)$$

Although we use matrix inversion here for convenience, the **IOC** could be found with other methods, which do not require an explicit matrix inversion, such as the method based on Cramer's rule.

Note that the vector, $\left(\begin{array}{c} u\;\cdot \;n\\ v\;\cdot \;n \end{array}\right)$, is the sum of inverted vectors $p_{i}^{\prime },\left(\begin{array}{c} \sum u\\ \sum v \end{array}\right)$, and the inversion of its mean (hva) is the harmonic vector average (**HVA**),

(3) $$HVA=\frac{k}{{\left|\left(\begin{array}{c} \sum u\\ \sum v \end{array}\right)\right|}^{2}}\;\left(\begin{array}{c} \sum u\\ \sum v \end{array}\right).$$
